# A retrospective evaluation of the clinical monitoring period prior to referral for glaucoma surgery with the emphasis on visual field test results

**DOI:** 10.1186/s12886-025-03925-z

**Published:** 2025-03-11

**Authors:** Jesper L. Hougaard, Boel Bengtsson

**Affiliations:** 1 Department of Clinical Sciences in Malmö, Ophthalmology, Lund University, Malmö, Sweden; 2 Department of Ophthalmology, Skåne University Hospital, Malmö, Sweden

**Keywords:** Referral, Monitoring, Rate of progression, Visual field, Intraocular pressure, Xen gel stent, Trabeculectomy, Surgery, Quality of life, Glaucoma

## Abstract

**Purpose:**

To survey the monitoring of patients who underwent glaucoma surgery with the purpose of identifying routines possibly delaying the referral process.

**Methods:**

We conducted an observational retrospective study of the 2-year period prior to referral of a cohort of patients who underwent trabeculectomy or Xen® Gel Stent implantation at Skåne University Hospital (SUS), Sweden. Data were retrieved from medical records; variables of particular interest were related to intraocular pressure (IOP) measurements and visual field (VF) tests.

**Results:**

Out of 330 patients who underwent surgery, 139 were included. There were 20 referring clinics in total; SUS and two clinics in private practice accounted for 55.4% of all referrals. Prior to referral, the most common number of VF tests per patient was three, and 43.2% (60/139) had ≥ 10 IOP measurements. According to the last VF test, 51.1% had lost > 50% of a full VF. During the 2-year period, 21.9% (28/128 with ≥ 2 VF tests) progressed from ≥ 50% to < 50% remaining of a full VF. The median rate of progression of VF damage was -6.8%/year in the 107 patients who had ≥ 3 VFs, and 67.3% (72/107) were projected, by extrapolation of the linear trend, to lose > 50% of a full VF if the referral had been postponed for 2 years. At the time of the last IOP measurement prior to referral, 84% (117/139) of the patients were on ≥ 3 IOP-lowering agents, and the IOP ranged from 11–45 mmHg, with a median of 20 mmHg.

**Conclusion:**

In general, in the 2-year period prior to referral for surgery, the number of IOP measurements was high, and the number of VF examinations seemed acceptable in most cases. Nevertheless, > 50% had advanced to severe VF loss and fast progression prior to referral. The IOP level is a known risk factor for disease progression that should be monitored at least by VF tests, but the frequent IOP measurements observed in our study, probably due to treatment changes, may have delayed the time to referral.

## Introduction

There are many papers reporting the outcome of surgical interventions in glaucoma patients, but few have been published regarding referrals for first-time glaucoma surgery or regarding the monitoring of patients and patient/disease-related characteristics during the time prior to referral for glaucoma surgery [1, 2]. The European Glaucoma Society [3] and the national Swedish Ophthalmological Society [4, 5] provide guidelines for monitoring glaucoma patients, including recommendations for the frequency of perimetry not to miss dangerously fast progression of visual field damage that risks affecting the patient’s vision-related quality of life (VR-QoL) within the expected lifetime. In such cases of fast disease progression, one should aim for a significant lowering of the intraocular pressure (IOP) by intensifying treatment, if possible, or refer for surgical intervention. Factors other than the progression of visual field (VF) loss, such as insufficient IOP reduction or intolerance to glaucoma treatment, may be the reason for the referral of patients for glaucoma surgery.

In the present survey, our primary focus was on reporting IOP measurements and VF test results and the number of examinations performed during a 2-year period prior to referral for glaucoma surgery. We also project the level of VF damage 2 and 5 years from the time of referral using extrapolation of the rate of VF progression prior to referral. Finally, we aimed to identify possible patient management routines that could predict the need for earlier referral of patients, especially in the era of safe trabeculectomy [6, 7] and minimally invasive bleb surgery, where the surgical risk is comparable to or possibly less than that of standard methods [8–10]. The effects of prereferral management on the effects of surgery, the effects of different surgical methods and a survey of clinical management over two years postoperatively will be reported separately.

## Methods

The present study was performed in accordance with the tenets of the Declaration of Helsinki, and the study was approved by the Swedish Ethical Review Authority. Owing to the retrospective nature of the study, patients were informed by postal mail about the study and the methods applied to collect data. The patients were asked to respond if they disapproved the use of their medical record data for the purpose of the current report. In addition, patients referred from clinics in private practice were contacted by a phone call from the principal investigator of the current project (JH) to provide oral consent. This latter procedure was requested by the administration of the private clinics.

We used a surgical coding register to identify all patients who underwent surgery with trabeculectomy or Xen® Gel Stent (Xen-45, Abbvie) from 1st October 2017 to 1st October 2019 at the Department of Ophthalmology, Skåne University Hospital (SUS) in Malmö, Sweden. Two trained glaucoma surgeons with experience in both surgical techniques performed the majority of preoperative visits at the surgical glaucoma unit at SUS or were involved in the decision to perform the surgery and in deciding the technique. Trabeculectomy was never combined with phakoemulsification. The indication for choosing the XEN gel stent as a stand-alone procedure or a trabeculectomy was not established in general or clear to the surgeons during the time of the study; the decision was made in agreement with the patient and did not follow a research protocol due to the retrospective study design. Retrospective data of interest from the patients’ records from 2 years prior to referral up to the date of referral were collected. Paper copies of patient records and VF test results were retrieved in cases where data were not accessible electronically. To limit the period of data collection to 2 years was chosen to ease the workload for the referring clinics.

The population of Skåne (Scania) County was approximately 1.34 million in 2017. The catchment area for SUS glaucoma surgery included two in-house glaucoma units, one located in Malmö and the other in Lund, departments of ophthalmology located at other community hospitals in Skåne, and ophthalmologists’ clinics in private practice in Skåne. Some patients were also referred from clinics outside the county. At the time of the data collection, a few of the referring community hospital departments within the county had smaller surgical glaucoma units, but none of these departments performed Xen® Gel Stent surgery. No clinics in private practice offered glaucoma surgery.

**Fig. 1:**
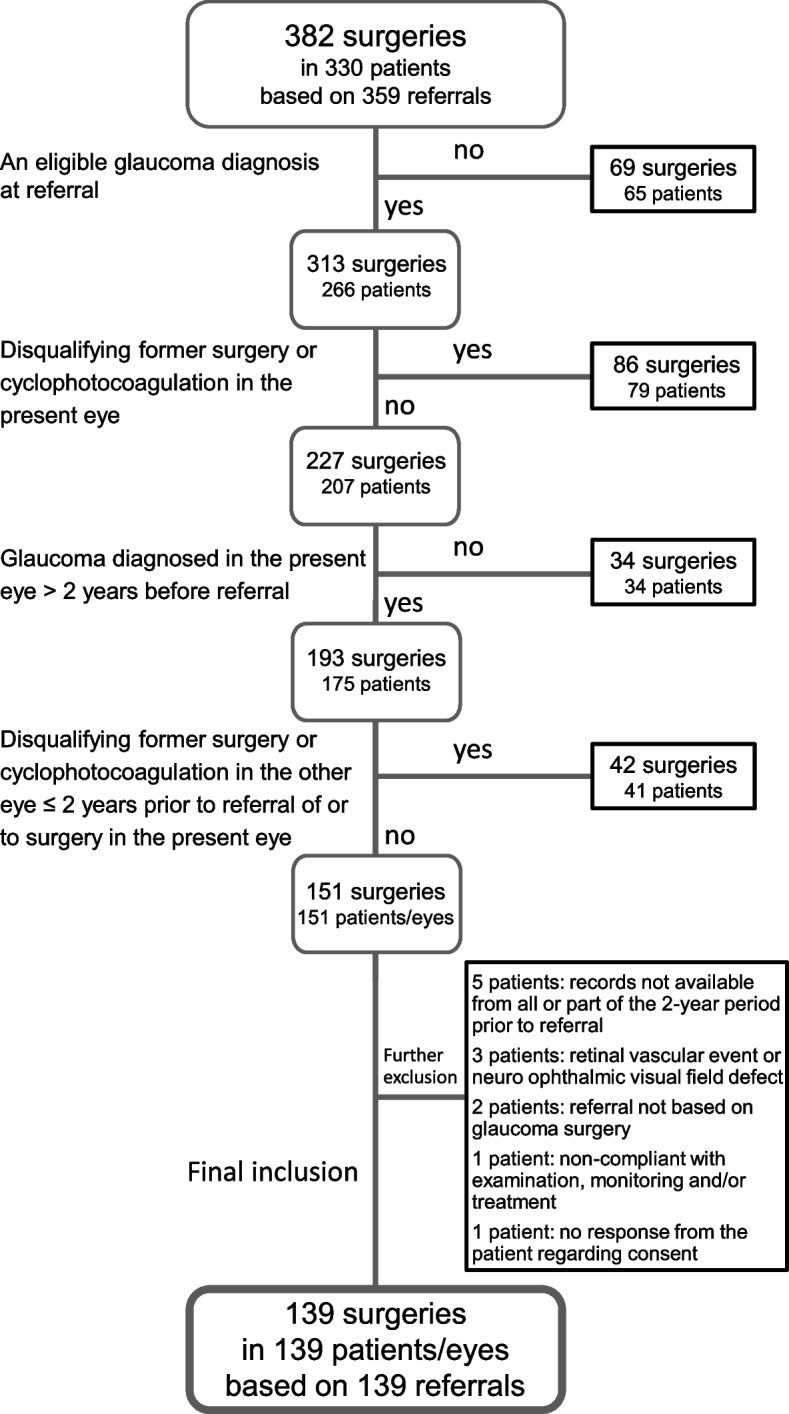
Flow chart of study inclusion and exclusion criteria. Among the 382 eligible surgeries, 139 eyes from 139 glaucoma patients were included. Eligible surgeries included trabeculectomy or Xen® Gel Stent (Xen-45, Abbvie) implantation performed during a 2-year period at the surgical glaucoma unit of Skåne University Hospital in Malmö, Sweden

Patients older than 18 years of age referred by an ophthalmologist were eligible. Given that the 2-year period prior to referral for glaucoma surgery was the focus of our survey, we wanted to be certain that the included eye of the patient had had a glaucoma diagnosis for at least this period and had not undergone any glaucoma surgery before referral. A flow chart (Fig. 1) shows the inclusion and exclusion criteria of patients for the final data analyses.

To be included, patients had to have one of the following glaucoma diagnoses: primary open angle glaucoma (POAG), pseudoexfoliative glaucoma (PEXG) or pigmentary glaucoma. The diagnosis was based on patient records of slit lamp findings, including a notation of a glaucomatous optic nerve head, which could be supported by optical coherence tomography or photography, and VF test results showing typical glaucomatous VF defects, which should be observed at least 2 years prior to referral, and the diagnosis was confirmed at the preoperative visit, typically at the surgical glaucoma unit. The majority of patients were tested with the HFA perimeter (Humphrey Field Analyzer, Carl Zeiss Meditec, Dublin, CA, USA). For these patients, we required the glaucoma hemifield test (GHT) to repeatedly be “outside normal limits” or the first test to be “borderline”, followed by tests “outside normal limits”. The VF defects were required to appear in the same area of the field. For the purpose of the current study, patients with findings of goniodysgenesis, juvenile glaucoma or mixed closed- and open-angle glaucoma pathogenesis reported during the last 2 years prior to referral were excluded.

Only eyes with no former IOP-lowering, corneal or intraocular surgery (except for uncomplicated phacoemulsification) or cyclophotocoagulation could be included. These kinds of surgeries were allowed in the fellow eyes but only if they were performed more than 2 years prior to the time of referral or IOP-lowering surgery of the present eye. Consequently, in a few cases, IOP-lowering surgery was not the first surgery for the patient but rather for the included eye of the patient. Figure 1 includes the criteria of the last steps of patient exclusion.

The reason for referral was defined as stated by the ophthalmologist. Intolerance to medical treatment was defined as any kind of local or systemic intolerance to one or more IOP-lowering substances. The number of IOP-lowering medical treatments used was defined as the total number of different substance groups used (betablockers, alpha-agonists, carbonic anhydrase inhibitors, prostaglandin analogues or pilocarpine).

During the period of interest, the patients were under care by different ophthalmologists, as well as ophthalmologically trained healthcare professionals at the referring community facilities and in private practices. Consequently, visual acuity, IOP and VF examinations were not standardised. Data on all IOPs and VF tests measured during the study period were retrieved from the patients’ records. The Visual Field Index (VFI) of the HFA expressing the VF as a percentage of a full field [11, 12] was chosen as the VF test outcome parameter. The VFI is not available for the other perimeters but can be approximated by roughly converting the VF mean defect (MD) to a percentage of the full MD scale value.

To be included, the VF tests had to be reliable. VFs were defined as reliable if the blind spot was visible in the greyscale map of the raw threshold values, if the gaze tracker did not support fixation difficulties in the few cases where the blind spot was not visible, if there were no signs of erratic VFs, for example, a “clover leaf pattern”, and if the proportion of false positive responses was less than 15%. VFs with a false positive proportion above 15% could be included if the threshold sensitivities were not abnormally high (without obvious “white scotomas” in the greyscale map of raw threshold values or not categorised with “abnormally high sensitivity” by the GHT).

### Statistics

One eye per patient was included. If a patient had two eligible eyes, the eye that had surgery first was included.

During the study period, the number of all IOP measurements was counted, and the mean and peak IOP values were calculated for all eyes. POAG patients were divided into two groups according to the median split of individual mean IOP values. Patients with treated IOP below the median of 18 mmHg were referred to as POAG patients with lower IOP, and those with IOP equal to or above the median were referred to as POAG patients with higher IOP. The VFI, or its equivalent, of the last VF test prior to referral and the time from that last VF test to referral were recorded and the number of VF tests taken during the 2-year period was counted. The VF rate of progression was calculated by linear regression analysis of the VFI over time. We required a minimum of 3 reliable VFs performed during the 2 years prior to referral to balance the concern of regression analysis uncertainty while keeping a representative number of patients for the study group. A minimum treatment goal could be that patients should keep at least 50% of the VF. Reports have suggested that patients’ VR-QoL starts to be affected when less than 50% of the VF in the best eye remains [13, 14]. A VFI of 50% corresponds to an MD of approximately −15 dB, depending on patient age.

The VF rate of progression trend was extrapolated to estimate VFI values 2 and 5 years ahead from the time of referral.

Normally distributed variables are described by the mean and 95% confidence interval (95% CI), and differences between variables were tested by t tests or one-way ANOVA with the post hoc Bonferroni correction to correct for Type 1 errors. Non-normally distributed variables are described by the mode, minimum and maximum (discrete data) or by the median and empirically derived 95% CI (continuous data), and differences between variables were tested by the Mann‒Whitney independent samples test or the Kruskal‒Wallis test with the post hoc Bonferroni correction to correct for Type 1 error. The Pearson chi-square test was used to analyse differences between categorical variables. Correlation between parameters were assessed by linear regression.

One single patient with the diagnosis of pigmentary glaucoma was not included in the statistical analyses of differences among patient diagnosis categories.

## Results

Out of 382 eligible surgeries in 330 patients, 139 eyes of 139 patients were included for further analyses (Fig. 1). Age, sex, laterality, preoperative lens status and glaucoma diagnoses are shown in Table 1.

**Table 1:** General and ocular characteristics at the time of referral for glaucoma surgery

**Number** (eyes/patients)	139/139
**Age** (years) [mean ± 1.96 × SD]	74.1 ± 16.0
**Sex** (female/male patients)	56.1%/43.9%
**Laterality** (right/left eye)	43.2%/56.8%
**Preoperative lens status:**	
Phakic/Pseudophakic	36.7%/63.3%
**Glaucoma diagnosis:**	
Primary open-angle glaucoma	57.6%^a^/54.0%^b^
Pseudoexfoliative glaucoma	41.7%^a^/45.3%^b^
Pigmentary glaucoma	0.7%^a^/0.7%^b^

^a^The diagnosis according to the referral

^b^The diagnosis according to a retrospective assessment

A large proportion (45.3%) of all included patients had PEXG. Trabeculectomy was performed in 41.0%, Xen® Gel Stent (Xen45) was performed in 41.7% and Xen® Gel Stent (Xen45) combined with phacoemulsification was performed in 17.3% of the included patients.

### Referrals

**Fig. 2:**
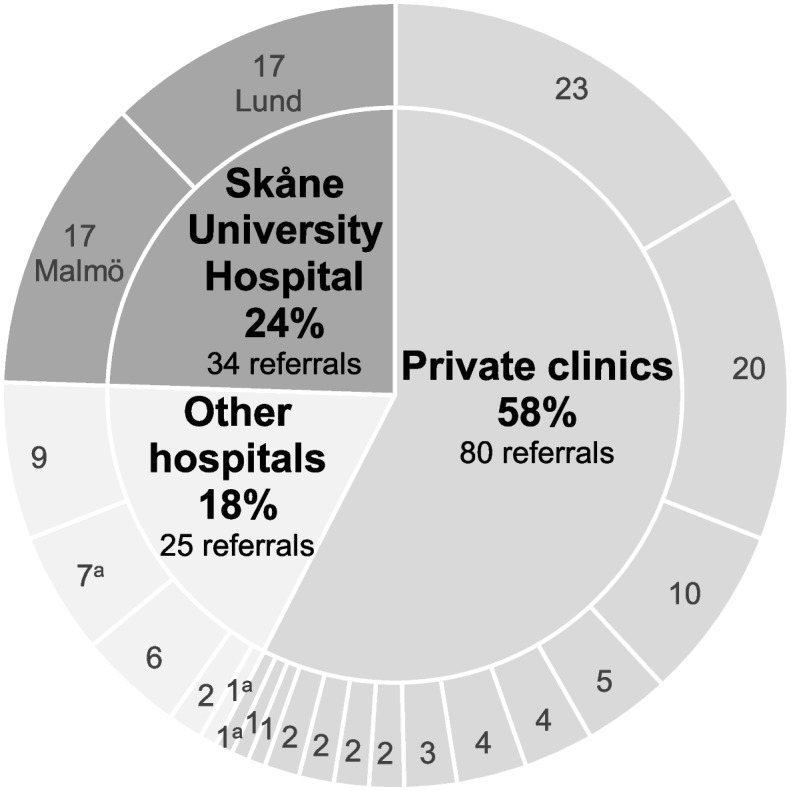
Referring units. The numbers of referrals (patients) from each of the 21 single referring units (14 clinics in private practice and 7 community hospital eye departments) are stated in the figure’s outer ring. Four referring units accounted for 55.4% of all 139 referrals. Most (58%) of the patients included were referred from a private clinic.^a^Referring units located outside Skåne County

The distributions of referrals among the referring units and referring institution categories are shown in Fig. 2.

**Fig. 3:**
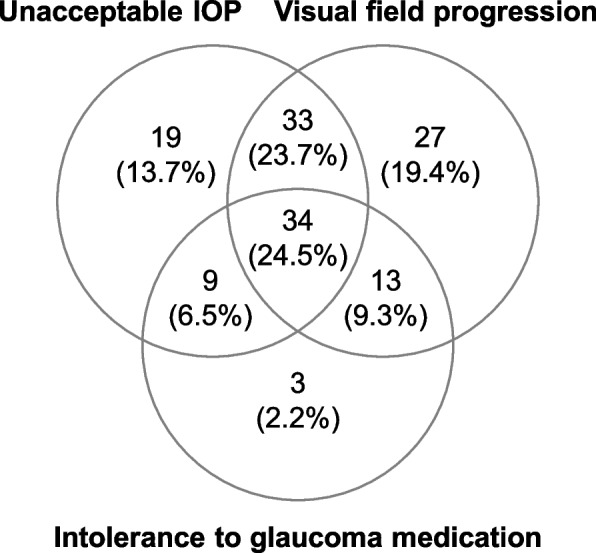
Reason for referral for glaucoma surgery. The numbers indicate the number of referrals (one per patient). Of the 139 included referrals/patients, one is not included in the diagram, as no obvious reason was stated in the referral, but all 139 referrals/patients are used when calculating the proportions. The most frequent reason for referral was a combination of an unacceptable level of IOP, visual field progression and intolerance to glaucoma medication stated in the referral and/or in the patient record(s). *IOP* intraocular pressure

Among all 21 referring units, most referrals came from the in-house SUS clinic and from two private settings. Together, these four units/three clinics accounted for 55.4% of all referrals. Most patients (74.8%) were referred for surgery in one eye, and 25.2% were referred for surgery in both eyes. A patient could have been managed at more than one clinic during the 2 years prior to referral, but the vast majority, 84.5% of patients, were managed at one and the same clinic. The single most frequent reason for referral, 19.4%, was VF progression followed by unacceptably high IOP, 13.7%, whereas intolerance to glaucoma medication was the single reason, accounting for only 2.2% of the cases (Fig. 3), but was noted in the referrals and/or patient records in 42.4% of all the referrals.

### Treatment of glaucoma

At the time of referral, 35.3%, 42.4%, and 6.5% of patients used three, four and a maximum of five IOP-lowering medical treatments, respectively. A smaller proportion (15.8%) used two or fewer IOP-lowering medical treatments. The proportion of patients in which treatment with pilocarpine or a systemic carbonic anhydrase inhibitor was ongoing or prescribed pending surgery was 25.9% and 20.9%, respectively. One or more treatments with laser trabeculoplasty were noted in the records of the majority (83.5%) of the patients.

### IOP- and VF-related parameters

The last measured IOP prior to referral ranged from 12.0 to 37.5 mmHg (95% confidence interval), with a median of 20.0 mmHg. At that last measurement, 18.7% of the patients had an IOP > 25 mmHg. The last IOP measurement before referral occurred within 1 day in 72.7% of the patients and between 32 to a maximum of 98 days in 6.5% of the patients. In 26.6% of the patients, the individual mean follow-up IOP was > 21 mmHg. The peak IOP was > 25 mmHg in 46.0% of the patients. Table 2 shows the IOP levels for different IOP measurement parameters before referral.

**Table 2:** Proportions of all 139 patients/eyes with different levels of IOP prior to referral

**Parameter levels**	**Parameter**		
	**Last IOP**^a^	**Mean IOP**^b^	**Peak IOP**^c^
>10 mmHg	100%	100%	100%
>15 mmHg	77.7%	83.5%	94.2%
>18 mmHg	60.4%	58.3%	81.3%
>21 mmHg	41.0%	26.6%	63.3%
>25 mmHg	18.7%	7.2%	46.0%

^a^The IOP at the last measurement prior to referral

^b^The average of all IOP measurements performed during the 2 years prior to referral

^c^The peak measurement among all IOP measurements performed during the 2 years prior to referral

*IOP* intraocular pressure

The number of IOP measurements ranged from 3 to 28, with the most frequent number being 8, and in 43.2% and 10.1% of patients, this number was ≥ 10 and ≥ 15, respectively. The peak IOP correlated moderately (*r* = 0.39) and significantly (*p* < 0.001) with the number of IOP measurements, whereas the individual mean IOP showed only a weak (*r* = 0.19) but significant (*p* = 0.042) correlation.

The vast majority, 84.2%, of the last IOPs measured prior to referral were performed by the gold standard Goldmann applanation tonometry. The specific use of Goldmann applanation tonometry was noted at every single measurement in 58.3% of patients. In patients managed at one and the same clinic during the period of interest, a mix of IOP measurement methods for two or more IOP measurements were used in 25.4%, 23.5% and 6.3%, respectively, of those referred from clinics in private practice, other community hospitals and the SUS.

At the last VF test before referral, more than half of the patients had lost more than 50% of their full field, with a VFI < 50% (Table 3).

**Table 3:** Proportion of all 139 patients/eyes with different last VFI values prior to referral

Parameter levels	Proportion
VFI value at the last visual field test	
< 10%	2.9%
< 30%	25.2%
**< 50%**	**51.1%**
< 70%	74.8%
< 90%	96.4%

*VFI *Visual Field Index

Most patients, 62.6%, had their last VF test before referral within 31 days; in 20.9% of the patients, this difference was > 3 months. The inability to perform VF tests was noted as the cause in the records for only one of these patients.

During the study period, the number of VF tests ranged from 1‒11, with the most frequent number being 3. In 9 of the 139 patients, only one VF test was performed.

**Fig. 4:**
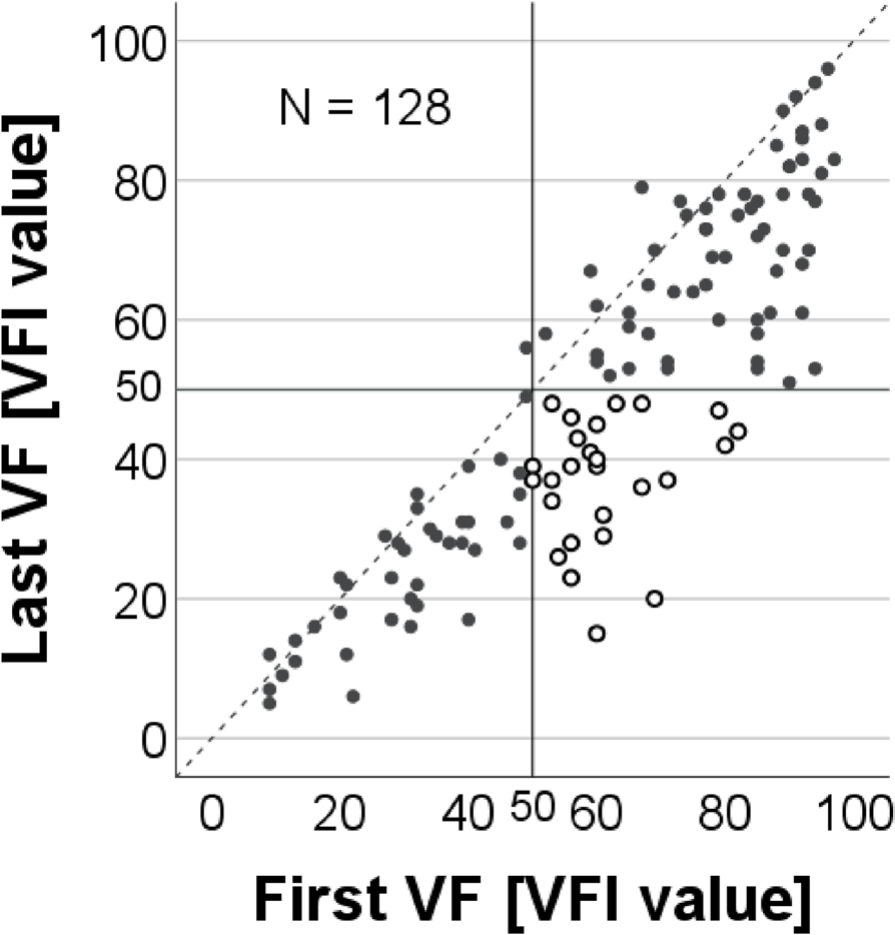
Visual field test results at the first and last tests during a 2-year period prior to referral for surgery. The results represent the 128 eyes/patients with at least 2 visual field (VF) tests during the period. The Visual Field Index (VFI) value expresses the percentage remaining of a full VF. The open circles represent the 21.9% (28/128) who progressed from ≥ 50% to < 50% remaining of a full VF prior to referral

Among the 128 patients with two or more reliable VF tests, 30.5% had lost more than 50% of the full VF at the first VF test within the study period, whereas 51.6% had lost more than 50% at the last VF test (Fig. 4).

For the 107 patients who had three or more reliable VF tests, the median rate of progression of VF damage was a loss of 6.8 percentage units per year, with the 95% CI ranging from −35.4 to 5.7 VFI value percentage units per year. If referral for surgery had been postponed for 2 and 5 years, the percentages of patients (n = 107) with a VF loss of more than 50% were projected to be 67.3% and 77.6%, respectively.

The HFA perimeter and the SITA test programs were used for all VF tests prior to referral in 125 (89.9%) of the 139 patients. The Octopus perimeter and the TOP programme (Haag-Streit International) and the Henson perimeter and the ZATA programme (Elektron Eye Technology/Topcon Healthcare) were used for all VF tests in 5 and 4 of the 139 patients, respectively, and with these two perimeters, the same test strategy was used for every single VF test performed. Five patients underwent VF tests with two different perimeters.

### Differences between referring institution categories

The last IOP measured before referral and the peak IOP during follow-up were significantly higher in patients referred from community hospital eye departments than in those from the SUS, and more VF examinations were performed in patients referred from the SUS than in those from clinics in private practice; for details, see Table 4.

**Table 4:**
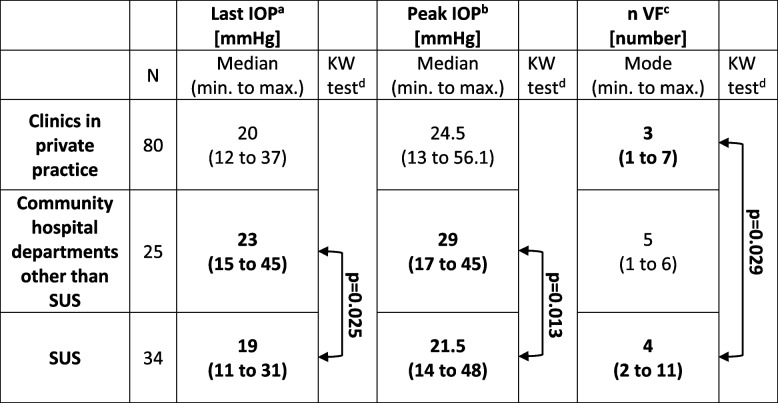
Parameters with significant differences between referring institution categories in 139 patients/eyes referred for glaucoma surgery

^a^The IOP at the last measurement prior to referral. ^b^The peak measurement among all IOP measurements performed during the 2 years prior to referral. ^c^Number of all visual field tests performed during the 2 years prior to referral. ^d^Kruskal‒Wallis test with Bonferroni‒corrected pairwise comparisons. *IOP* intraocular pressure, *SUS* Skåne University Hospital, the in-house university hospital eye department

### Differences between patients with POAG with lower and higher IOP and PEXG

In terms of the median split, POAG patients with lower IOP had lower last, peak and mean IOP than did PEXG patients, and they were also younger than PEXG and POAG patients with higher IOP (Table 5). There were no statistically significant differences in these parameters between POAG patients with higher IOP and PEXG patients.

**Table 5:**
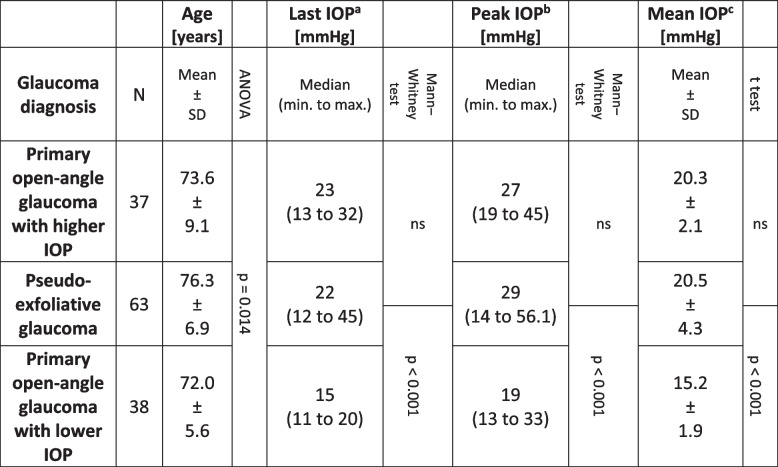
Parameters with significant differences between glaucoma diagnosis categories in 138 patients/eyes referred for glaucoma surgery

Note: n ≠ 139, as a single patient with pigmentary glaucoma was excluded from the analyses

^a^The IOP at the last measurement prior to referral

^b^The peak measurement among all IOP measurements performed during the 2 years prior to referral

^c^The average of all IOP measurements performed during the 2 years prior to referral

*IOP* Intraocular pressure, *ns* Non-significant

The median time from the last VF test to the time of referral was shorter in POAG patients with lower IOP than in POAG patients with higher IOP and PEXG patients (2, 9 and 34 days, respectively). The most common number of VF tests during the study period was four in both patients with POAG with lower IOP and PEXG, but the frequency distributions of the number of VF tests differed significantly with somewhat more VF tests performed in patients with POAG with lower IOP (p = 0.046).

The number of IOP-lowering medical treatments used in the three different patient groups did not differ significantly. The proportion of patients in whom treatment with a systemic carbonic anhydrase inhibitor was ongoing or prescribed pending surgery at the time of referral was 31.7%, 19.0% and 5.3% in patients with PEXG, POAG patients with higher IOP and those with lower IOP, respectively, which was statistically significantly greater in PEXG patients than in POAG patients with lower IOP (p = 0.002).

The median visual acuity, best corrected and/or with a stenopeic pinhole, closest to the referral point, was somewhat worse in patients with PEXG than in POAG patients with higher IOP (0.7 and 0.8, respectively; p = 0.007) and POAG patients with lower IOP (0.7 and 0.75, respectively).

## Discussion

In the present retrospective study, we surveyed the monitoring of patients during the 2 years prior to referral for their first glaucoma surgery. All surgeries were performed at Skåne University Hospital (SUS), which is located in southern Sweden. In Sweden, patients are easy to trace because of the social security number register and because of helpful referring clinics. The majority of the included patients were referred from clinics in private practice, similar to a previous European study [1].

Reports have suggested that patients’ VR-QoL starts to be affected when less than 50% of the VF in the best eye remains [13, 14]. The European Glaucoma Society states that the overall goal of glaucoma management is to promote the best possible well-being and quality of life with minimal glaucoma induced visual disability, and that; “it is the extent of binocular VF or the field of the better eye that largely determines the patient’s (vision-related) quality of life, while the rates of progression of each eye separately are needed to determine treatment” [3]. In our study of the one eye per patient that had the glaucoma surgery performed, we proposed that a minimum treatment goal could be that patients should keep at least 50% of the VF. Already at the time of the first VF test, 39/128 (31%) of the patients had lost more than 50% of a full VF, and this proportion increased to 66/128 (52%) of the patients at the last VF test before referral. At that last VF test the loss was somewhat greater than that reported average VF mean deviation of −13.3 dB in “old” European countries [1]. Furthermore, 72/107 (67.3%) of the patients with three or more VF tests were projected to have a VF loss of worse than 50% if the referral had been postponed by 2 years.

Deciding whether to perform surgery or not and what method/technique to choose should be handled with care by experienced glaucoma surgeons and in agreement with the patient, as surgery always involves a risk of complications and requires postoperative patient compliance. However, the modern trabeculectomy technique and XEN-45 stent implantation, the most frequently used methods/techniques at our surgical department, have been reported to be effective and to have an acceptable safety profile [10, 15–19].

In our survey, the patients were followed on average with a fair number of VF tests if managed in an ordinary glaucoma clinic, but more frequent field testing would most likely have been able to detect fast progression and would have led to earlier referral for surgery.

Almost 22% of all patients did not have any VF test in the last 3 months before referral. Even if the focus may be on treating a high IOP, it is not optimal for the preoperative evaluation of referred patients whose VF status is not up to date, and the exact level of VF damage is also important for the surgeon to be able to choose between a trabeculectomy and other surgical techniques, as more advanced VF damage often favours trabeculectomy. A VF test is, for practical reasons, unfortunately not a part of the standard examination routine at the preoperative visit at many surgical glaucoma units.

Not surprisingly, the last IOP prior to referral was quite high in a rather large proportion of the study population. The significantly higher IOP level in patients referred from community hospital eye departments other than the SUS could be related to the fact that three out of five of these departments performed glaucoma surgery at the time of the present study but included a limited number of patients, who were likely elective surgeries. During follow-up, 27% of all referred patients had an individual mean IOP above 21 mmHg, and 43% of all patients had 10 or more IOP measurements performed during these 2 years. With our study design, we cannot exclude that this high number of IOP measurements may have been performed routinely during visits for a cause other than glaucoma and/or a competing condition or may have been due to patient reluctance to surgery, but in general, the high number was probably explained by several attempts to regulate the IOP by changing eye drops, performing laser trabeculoplasty or trying to test different eye drops to avoid local or systemic side effects. In support of this assumption, most patients were recorded at the visit where referral was decided to use several IOP-lowering medications, and local or systemic intolerance was noted in the referral or in the patient records in approximately 42% of patients. Laser trabeculoplasty is often an effective treatment for lowering the IOP [20, 21], and it can be repeated once effective. Nevertheless, it could be argued that repeated laser trabeculoplasty should be used only in patients with more advanced disease and a fast rate of progression of VF defects as a complement to IOP-lowering drugs as a temporary IOP-lowering treatment during the time from referral to surgery [2]. In our heterogeneous study, the number of IOP measurements performed prior to referral was not significantly correlated with the rate of progression of VF damage but rather with the level of IOP. However, the high number of IOP measurements observed seems intuitively much greater than that of glaucoma patients not in need of glaucoma surgery, and if this was a result of several attempts to change or add to treatment and/or perform laser treatments in patients at risk of developing serious visual field damage, it could postpone the time of referral for surgery unnecessarily. The observed but also the projected proportions of patients progressing to lose more than 50% of a full VF in our survey could also support referral at an earlier point in time. A delay in referring patients for glaucoma surgery may not be a specific issue for Sweden; the impression from panel discussions at international meetings seems to confirm this problem. In Sweden, glaucoma surgery at community hospital eye departments including the SUS, is free of charge for patients.

PEXG patients typically have higher IOP than POAG patients do [22–24], and higher IOP is a well-known and strong risk factor for fast progression [25, 26]. Therefore, it is not surprising that PEXG patients have a faster rate of progression of VF damage than POAG patients do [27, 28]. In the current survey, the median (95% CI) rate of progression of VF damage for patients with three or more VF tests was quite fast, −6.8 percentage units (−35.4 to 5.7) per year, and the lack of a significant difference between the diagnosis groups in this rate highlights the selection of patients in need of surgery.

Although the IOP level is the target of treatment and a known risk factor for development of and progressing of glaucoma its role in management is not straight forward as patients with lower pressures may progress while some patients with higher pressures will not progress and this due to other factors than merely the IOP. This fact makes monitoring the individual patient’s VF with an adequate number of VFs over time [29] to reduce the impact of normal test–retest variability on the results very important to approximate the IOP level that slow down, or even better, stabilize the glaucoma disease. Glaucoma surgery, if successful, may help the patient to reach that individual IOP level and reduce or completely eliminate medical treatment. Perimetry is the better current clinically applicable test to monitor the glaucoma disease progression, but its correlation to VR-QoL is not ideal. VF and VR-QoL are causally related, but the relation is complex, at least partially because methods of assessing VR-QoL and VF are both noisy. Figure 4 illustrate such VF variability problems as a few patients seems to get better over time when progression is evaluated based on only two VFs. Even if the baseline level of VF damage may predict the patients risk of going blind during their lifetime [30] Fig. 4 also shows that severe damage of the VF may develop over a short period disregarding the amount of initial VF loss and apart from considering risk factors for progression. Risks of falls and driving accidents have been reported to be higher in glaucoma patients than in controls [31, 32] and often patients do not link these accidents to their glaucoma disease as they are not aware of their visual disability most likely explained by the filling-in effect of VF defects in the visual cortex. Although VR-QoL measuring instruments in some cases may be affected also in earlier stages of glaucoma, it is important to monitor the VF to be able to initiate or change treatment with the aim of preventing patients getting VR-QoL related problems.

Even if gold standard Goldmann applanation tonometry was used for measuring the IOP in most patients, the use of a mix of methods was quite common in both private clinics and community hospital eye departments other than the SUS. The European Glaucoma Society guidelines state that different methods should not be used interchangeably during the follow-up of glaucoma patients [3] because of measurement variation between methods.

The SITA threshold test strategy implemented in the HFA was by far the most commonly used method, but in private clinics using Octopus perimetry the TOP test strategy was used. The TOP test strategy is a fast VF test intended for patients who have difficulties performing a VF test [33]. The test strategy has been reported to have large test‒retest variability, which makes it less capable of detecting VF progression [34]. The Octopus perimeter provides considerably more reliable VF test strategies than the TOP strategy.

The design of this survey did not allow us to evaluate exactly how long all the patients had had their glaucoma diagnosis and what the VF result was at diagnosis, which could be of interest to the interpretation of the results. Our report is based on data retrieved from different types of institutions and the inclusion of different patient subgroups regarding glaucoma diagnosis, reason for referral, IOP and VF damage level, and rate of progression, which limits the ability to analyse differences between these subgroups. Differences between measurement methods and examinations applied at different clinics may affect the results somewhat. The retrospective study design could also lead to diagnostic uncertainties. In the present study, we defined POAG patients with lower or higher IOPs using the median split of the treated IOP. We also had to accept some uncertainty when evaluating the progression of VF damage based on 2 VFs only and when calculating the rate of progression of VF damage using three or more VF tests.

Despite the limitations, we believe that our survey provides important and clinically relevant insights into the monitoring patterns of patients referred for glaucoma surgery and adds clinically applicable ideas concerning when to refer a patient for glaucoma surgery. Representing the current population, results will be reported separately on the effects of, for example, prereferral management on the effects of surgery and visual field test results 2 years postoperatively.

In conclusion, the number of IOP measurements was high and the number of VF tests performed seemed generally acceptable during the study period prior to referral for glaucoma surgery. During the 2-year study period prior to the referral, patients had fast rate of progression of VF defects, and of those with 2 or more VFs, 22% progressed to a loss of more than half of a full VF. Referring patients with a VF older than 3 months prior to referral is not ideal, and the use of different IOP measurement methods during follow-up should be avoided for obvious reasons, especially in patients with or at risk of developing severe VF damage. The IOP level is a known risk factor for disease progression that should be monitored at least by VF tests, but the frequent IOP measurements observed in our study, probably due to treatment changes, may have delayed the time to referral. More effective methods and strategies to safely treat and monitor the glaucoma disease to prevent visual disability and reduced quality of life would likely improve the management of glaucoma patients at risk of developing severe VF damage affecting VR-QoL.

## Data Availability

The datasets generated and/or analyzed during the current study are not publicly available due to limitations of the ethical approval involving the patient data and anonymity but are available from the corresponding author on reasonable request.

## Abbreviations

SUS: Skåne University Hospital
IOP: Intraocular pressure
VF: Visual field
VR-QoL: Vision-related quality of life
POAG: Primary open angle glaucoma
PEXG: Pseudoexfoliative glaucoma
HFA: Humphrey Field Analyzer
GHT: Glaucoma Hemifield Test
VFI: Visual Field Index
MD: Mean deviation
95% CI: 95% Confidence interval

## References

[1] Hollo G, Schmidl D, Hommer A. Referral for first glaucoma surgery in Europe, the ReF-GS study. Eur J Ophthalmol. 2019;29(4):406–16. 30101618 10.1177/1120672118791937

[2] Sturmer JPE, Faschinger C. Do we perform glaucoma surgery too late? Klin Monbl Augenheilkd. 2018;235(11):1269–77. 28837978 10.1055/s-0043-115902

[3] European Glaucoma Society Terminology and Guidelines for Glaucoma, 5th Edition. Br J Ophthalmol. 2021;105(Suppl 1):1–169. 10.1136/bjophthalmol-2021-egsguidelines. 10.1136/bjophthalmol-2021-egsguidelines 34675001

[4] Heijl A, Alm A, Bengtsson B, Bergstrom A, Calissendorff B, Lindblom B, Linden C. Swedish Ophthalmological Society: The Glaucoma Guidelines of the Swedish Ophthalmological Society. Acta Ophthalmol Suppl (Oxf ). 2012;251:1–40. 10.1111/j.1755-3768.2012.02415.x 23279889

[5] Johannesson G, Stille U, Taube AB, Karlsson M, Kalaboukhova L, Bergstrom A, Peters D, Linden C. Guidelines for the management of open-angle glaucoma: National Program Area Eye Diseases. National Working Group Glaucoma Acta Ophthalmol. 2024;102(2):135–50. 10.1111/aos.16599 38164112

[6] Khaw PT, Chiang M, Shah P, Sii F, Lockwood A, Khalili A. Enhanced Trabeculectomy: The Moorfields Safer Surgery System. Dev Ophthalmol. 2017;59:15–35. 28442684 10.1159/000458483

[7] King AJ, Fernie G, Hudson J, Kernohan A, Azuara-Blanco A, Burr J, Homer T, Shabaninejad H, Sparrow JM, Garway-Heath D, et al. Primary trabeculectomy versus primary glaucoma eye drops for newly diagnosed advanced glaucoma: TAGS RCT. Health Technol Assess. 2021;25(72):1–158. 34854808 10.3310/hta25720

[8] Birnbaum FA, Neeson C, Sola-Del Valle D. Microinvasive Glaucoma Surgery: An Evidence-Based Review. Semin Ophthalmol. 2021;36(8):772–86. 34297650 10.1080/08820538.2021.1903513

[9] Ahmed IIK, Sadruddin O, Panarelli JF. Subconjunctival filtration in evolution: current evidence on MicroShunt implantation for treating patients with glaucoma. Eye Vis (Lond). 2023;10(1):10. 36859515 10.1186/s40662-022-00322-1 PMC9979478

[10] Traverso CE, Carassa RG, Fea AM, Figus M, Astarita C, Piergentili B, Vera V, Gandolfi S. Effectiveness and Safety of Xen Gel Stent in Glaucoma Surgery: A Systematic Review of the Literature. J Clin Med. 2023;12(16):5339. 10.3390/jcm12165339. 10.3390/jcm12165339 PMC10455777 37629380

[11] Bengtsson B, Heijl A. A visual field index for calculation of glaucoma rate of progression. Am J Ophthalmol. 2008;145(2):343–53. 18078852 10.1016/j.ajo.2007.09.038

[12] Heijl A, Patella V, Bengtsson B. Effective Perimetry. 4th ed. Dublin: Carl Zeiss Meditec, Inc.; 2012.

[13] Peters D, Heijl A, Brenner L, Bengtsson B. Visual impairment and vision-related quality of life in the Early Manifest Glaucoma Trial after 20 years of follow-up. Acta Ophthalmol. 2015;93(8):745–52. 26382936 10.1111/aos.12839 PMC5014208

[14] Jones L, Bryan SR, Crabb DP. Gradually Then Suddenly? Decline in Vision-Related Quality of Life as Glaucoma Worsens. J Ophthalmol. 2017;2017:1621640. 28469940 10.1155/2017/1621640 PMC5392404

[15] Kirwan JF, Lockwood AJ, Shah P, Macleod A, Broadway DC, King AJ, McNaught AI, Agrawal P. Trabeculectomy Outcomes Group Audit Study G: Trabeculectomy in the 21st century: a multicenter analysis. Ophthalmology. 2013;120(12):2532–9. 24070811 10.1016/j.ophtha.2013.07.049

[16] King AJ, Hudson J, Azuara-Blanco A, Burr J, Kernohan A, Homer T, Shabaninejad H, Sparrow JM, Garway-Heath D, Barton K, et al. Evaluating Primary Treatment for People with Advanced Glaucoma: Five-Year Results of the Treatment of Advanced Glaucoma Study. Ophthalmology. 2024;131(7):759–70. 38199528 10.1016/j.ophtha.2024.01.007 PMC11190021

[17] Rauchegger T, Krause SM, Nowosielski Y, Huber AL, Willeit P, Schmid E, Teuchner B. Three-year clinical outcome of XEN45 Gel Stent implantation versus trabeculectomy in patients with open angle glaucoma. Eye (Lond). 2024;38(10):1908–16. 38548944 10.1038/s41433-024-03042-z PMC11226636

[18] Arnould L, Balsat E, Hashimoto Y, White A, Kong G, Dunn H, Fan L, Gabrielle PH, Bron AM, Creuzot-Garcher CP, Lawlor M. Two-year outcomes of Xen 45 gel stent implantation in patients with open-angle glaucoma: real-world data from the Fight Glaucoma Blindness registry. Br J Ophthalmol. 2024;108(12):1672–8. 10.1136/bjo-2023-325077. 10.1136/bjo-2023-325077 PMC11671995 38789132

[19] Swaminathan SS, Jammal AA, Medeiros FA, Gedde SJ; Primary Tube Versus Trabeculectomy Study Group. Visual Field Outcomes in the Primary Tube Versus Trabeculectomy Study. Ophthalmology. 2024;131(10):1157–63. 10.1016/j.ophtha.2024.03.026. 10.1016/j.ophtha.2024.03.026 PMC11416337 38582154

[20] Rasmuson E, Bengtsson B, Linden C, Heijl A, Aspberg J, Andersson-Geimer S, Johannesson G. Long-term follow-up of laser trabeculoplasty in multi-treated glaucoma patients. Acta Ophthalmol. 2024;102(2):179–85. 37278271 10.1111/aos.15718

[21] Gazzard G, Konstantakopoulou E, Garway-Heath D, Adeleke M, Vickerstaff V, Ambler G, Hunter R, Bunce C, Nathwani N, Barton K. LiGHT Trial Study Group: Laser in Glaucoma and Ocular Hypertension (LiGHT) Trial: Six-Year Results of Primary Selective Laser Trabeculoplasty versus Eye Drops for the Treatment of Glaucoma and Ocular Hypertension. Ophthalmology. 2023;130(2):139–51. 36122660 10.1016/j.ophtha.2022.09.009

[22] Anastasopoulos E, Founti P, Topouzis F. Update on pseudoexfoliation syndrome pathogenesis and associations with intraocular pressure, glaucoma and systemic diseases. Curr Opin Ophthalmol. 2015;26(2):82–9. 25594764 10.1097/ICU.0000000000000132

[23] Vesti E, Kivela T. Exfoliation syndrome and exfoliation glaucoma. Prog Retin Eye Res. 2000;19(3):345–68. 10749381 10.1016/s1350-9462(99)00019-1

[24] Häkkinen M, Ekström C. Distribution of intraocular pressure in a Swedish population. Ups J Med Sci. 2022;127. 10.48101/ujms.v127.8829. 10.48101/ujms.v127.8829 PMC9607869 36337274

[25] Founti P, Bunce C, Khawaja AP, Dore CJ, Mohamed-Noriega J, Garway-Heath DF. United Kingdom Glaucoma Treatment Study Group: Risk Factors for Visual Field Deterioration in the United Kingdom Glaucoma Treatment Study. Ophthalmology. 2020;127(12):1642–51. 32540325 10.1016/j.ophtha.2020.06.009

[26] Leske MC, Heijl A, Hyman L, Bengtsson B, Dong L, Yang Z. EMGT Group: Predictors of long-term progression in the Early Manifest Glaucoma Trial. Ophthalmology. 2007;114(11):1965–72. 17628686 10.1016/j.ophtha.2007.03.016

[27] Heijl A, Bengtsson B, Hyman L, Leske MC. Early Manifest Glaucoma Trial Group: Natural history of open-angle glaucoma. Ophthalmology. 2009;116(12):2271–6. 19854514 10.1016/j.ophtha.2009.06.042

[28] De Moraes CG, Liebmann JM, Liebmann CA, Susanna R Jr, Tello C, Ritch R. Visual field progression outcomes in glaucoma subtypes. Acta Ophthalmol. 2013;91(3):288–93. 21974913 10.1111/j.1755-3768.2011.02260.x

[29] Chauhan BC, Garway-Heath DF, Goni FJ, Rossetti L, Bengtsson B, Viswanathan AC, Heijl A. Practical recommendations for measuring rates of visual field change in glaucoma. Br J Ophthalmol. 2008;92(4):569–73. 18211935 10.1136/bjo.2007.135012 PMC2564806

[30] Peters D, Bengtsson B, Heijl A. Factors associated with lifetime risk of open-angle glaucoma blindness. Acta Ophthalmol. 2014;92(5):421–5. 23837818 10.1111/aos.12203

[31] Haymes SA, Leblanc RP, Nicolela MT, Chiasson LA, Chauhan BC. Risk of falls and motor vehicle collisions in glaucoma. Invest Ophthalmol Vis Sci. 2007;48(3):1149–55. 17325158 10.1167/iovs.06-0886

[32] Montana CL, Bhorade AM. Glaucoma and quality of life: fall and driving risk. Curr Opin Ophthalmol. 2018;29(2):135–40. 29266021 10.1097/ICU.0000000000000455

[33] Racette L, Fischer M, Bebie H, Holló G, Johnson C, Matsumoto C. Visual Field Digest - A guide to perimetry and the Octopus perimeter. 8th ed. Köniz: Haag-Streit AG; 2019. https://haag-streit.com/2%20Products/Speciality%20diagnostics/Perimetry/Category%20assets/Books/HS_perimetry_br_xxx_visual_field_digest_8th_en.pdf.

[34] Hollo G. Influence of Test Strategy on Octopus Perimeter Cluster Mean Defect Values: Adaptive Bracketing Normal Strategy Versus Tendency-oriented Perimetry. J Glaucoma. 2016;25(10):830–4. 27300642 10.1097/IJG.0000000000000456

